# Assignment of sigma factors of RNA polymerase to promoters in *Corynebacterium glutamicum*

**DOI:** 10.1186/s13568-017-0436-8

**Published:** 2017-06-24

**Authors:** Hana Dostálová, Jiří Holátko, Tobias Busche, Lenka Rucká, Andrey Rapoport, Petr Halada, Jan Nešvera, Jörn Kalinowski, Miroslav Pátek

**Affiliations:** 1 Institute of Microbiology of the CAS, v. v. i., Vídeňská 1083, 14220 Prague 4, Czech Republic; 2 Center for Biotechnology, Bielefeld University, 33594 Bielefeld, Germany

**Keywords:** *Corynebacterium glutamicum*, Promoter, Sigma factor, In vitro transcription, RNA polymerase

## Abstract

*Corynebacterium glutamicum* is an important industrial producer of various amino acids and other metabolites. The *C. glutamicum* genome encodes seven sigma subunits (factors) of RNA polymerase: the primary sigma factor SigA (σ^A^), the primary-like σ^B^ and five alternative sigma factors (σ^C^, σ^D^, σ^E^, σ^H^ and σ^M^). We have developed in vitro and in vivo methods to assign particular sigma factors to individual promoters of different classes. In vitro transcription assays and measurements of promoter activity using the overexpression of a single sigma factor gene and the transcriptional fusion of the promoter to the *gfp*uv reporter gene enabled us to reliably define the sigma factor dependency of promoters. To document the strengths of these methods, we tested examples of respective promoters for each *C. glutamicum* sigma factor. Promoters of the *rshA* (anti-sigma for σ^H^) and *trxB1* (thioredoxin) genes were found to be σ^H^-dependent, whereas the promoter of the *sigB* gene (sigma factor σ^B^) was σ^E^- and σ^H^-dependent. It was confirmed that the promoter of the *cg2556* gene (iron-regulated membrane protein) is σ^C^-dependent as suggested recently by other authors. The promoter of *cmt1* (trehalose corynemycolyl transferase) was found to be clearly σ^D^-dependent. No σ^M^-dependent promoter was identified. The typical housekeeping promoter P2*sigA* (sigma factor σ^A^) was proven to be σ^A^-dependent but also recognized by σ^B^. Similarly, the promoter of *fba* (fructose-1,6-bisphosphate aldolase) was confirmed to be σ^B^-dependent but also functional with σ^A^. The study provided demonstrations of the broad applicability of the developed methods and produced original data on the analyzed promoters.

**Electronic supplementary material:**

The online version of this article (doi:10.1186/s13568-017-0436-8) contains supplementary material, which is available to authorized users.

## Introduction

The multisubunit RNA polymerase (RNAP) holoenzyme in bacteria consists of core enzyme (2α, β, β′ and ω subunits) and a dissociable σ subunit (σ factor) that recognizes specific promoter sequences. Sigma factors are thus key regulatory elements that control different classes of promoters and activate expression of the respective groups of genes (regulons or sigmulons). Bacterial cells adapt in this way to changes in nutritional and environmental conditions. Bacteria typically possess a primary σ factor that is responsible for the transcription of housekeeping genes and a variable number of alternative σ factors that enable the cell to cope with various environmental stimuli. Since the activities of the different holo-RNAPs and the respective promoters orchestrate the cell metabolism in complex responses to various nutrition, growth and stress conditions, engineering σ factors has recently become a promising field in biotechnology and synthetic biology, particularly for the development of synthetic transcriptional control (Rhodius et al. 2013; Tripathi et al. 2014).

*Corynebacterium glutamicum* is a Gram-positive non-pathogenic soil bacterium used particularly for the industrial production of l-amino acids. The existing large toolbox for the genetic and metabolic engineering of *C. glutamicum* (Nešvera and Pátek 2011) enabled the construction of *C. glutamicum* producers of amino acids, carboxylic acids, alcohols, amines, polymers and biofuels as well as the use of alternative carbon sources like organic acids, pentoses, glycerol, starch and cellulose (Becker and Wittmann 2012). The *C. glutamicum* genome encodes seven sigma subunits of RNAP: the primary sigma factor σ^A^, the alternative primary-like σ^B^ and five other alternative σ factors with extracytoplasmic functions (ECF) (σ^C^, σ^D^, σ^E^, σ^H^ and σ^M^) (for a review, see Pátek and Nešvera 2011).

*Corynebacterium glutamicum* σ^A^ is an essential primary σ factor that directs the transcription of the majority of genes expressed during exponential growth which are termed “housekeeping” or “vegetative”. The promoters are usually considered housekeeping (σ^A^-dependent) if their −35 and −10 promoter sequences match the generally accepted consensus of housekeeping promoters. The consensus sequence of σ^A^-dependent promoters (−35 ttgnca and −10 TAnnnT) was deduced from a large number of defined promoters (Pátek and Nešvera 2011; Pfeifer-Sancar et al. 2013) which are believed to be σ^A^-dependent.

σ^B^ is a non-essential primary-like σ factor that is present in *C. glutamicum* cells in the highest levels at the transition and in the early stationary phase (Larisch et al. 2007). It is involved in responses to various stresses such as acid and heat stress and oxygen deprivation (Ehira et al. 2008; Halgasova et al. 2002). In addition to its involvement in stress-protective functions, σ^B^ drives the transcription of the genes active in glucose utilization during exponential growth (Ehira et al. 2008). σ^B^ can thus be considered to be a σ factor for slow growth and general stress conditions and another σ factor that recognizes some housekeeping promoters in the exponential growth phase. Only a few σ^B^-dependent promoters (13 in the review of Pátek and Nešvera 2011) have been localized and their key sequences were found to be essentially indistinguishable from the consensus sequences of σ^A^-dependent promoters.

σ^H^ is the most studied *C. glutamicum* ECF sigma factor, which controls a transcriptional regulatory network enabling the *C. glutamicum* cell to respond to temperature, oxidative and growth-phase induced stresses (Busche et al. 2012; Ehira et al. 2009; Toyoda and Inui 2016b; Toyoda et al. 2015). The consensus sequence −35 g/tGGAAt and −10 t/cGTTgaa was defined (Busche et al. 2012; Ehira et al. 2009) based on the 45 proposed σ^H^-regulated promoters.

*Corynebacterium glutamicum* σ^E^ was found to be involved in heat and cell surface stress response (Park et al. 2008). We have recently shown that the promoters P1*clgR*, P2*dnaK* and P2*dnaJ2* are recognized by both σ^E^ and σ^H^ (Šilar et al. 2016). This indicates that there is a certain overlap in promoter recognition specificity for σ^H^ and σ^E^. The consensus sequence of σ^E^-specific promoters has not been determined yet.

The σ^C^ regulon that is induced in response to defects in aerobic respiration has been recently described (Toyoda and Inui 2016a). Eight σ^C^-specific promoters were found, and their consensus sequence was defined as −35 GGAAAC and −10 CGACT.

A group of genes involved in oxidative stress response was found to be σ^M^-dependent (Nakunst et al. 2007). Some of these genes were found to be σ^H^-dependent in another study (Ehira et al. 2009). It therefore needs to be elucidated whether σ^H^ and σ^M^ are members of a regulatory cascade or if their recognition specificities overlap. No σ^D^-dependent genes and σ^D^-specific promoters have been described yet.

It was found that the overexpression of the *C. glutamicum sigH* gene resulted in enhanced riboflavin biosynthesis and its excretion to the medium (Taniguchi and Wendisch 2015; Toyoda et al. 2015). Further advances in the use of σ factor manipulations are still hampered in *C. glutamicum* by limited knowledge of the mechanisms of σ factor regulations, as well as by current lack of reliable methods for identifying the target promoters for various σ factors.

Knowledge of the recognition specificity of σ factors and assignment of σ factors to particular promoters is necessary to modulate the effects of sigma factors on the production of specific metabolites. In addition to manipulating sigma factors, the construction of artificial promoters recognized by specific σ factors is a promising strategy for modulating gene expression and improving the production of selected metabolites (Pátek et al. 2013). A self-inducible σ^B^-dependent *C. glutamicum* promoter has recently been developed which can be useful for the production of metabolites in the stationary growth phase (Kim et al. 2016).

Multiple promoters upstream of many bacterial genes pose another challenge to their classification. The genes have frequently two or more promoters, which can overlap. Two overlapping promoters can be controlled by different sigma factors. Moreover, some promoters are recognized by two or even more sigma factors. As a result, determining which regulon the gene belongs to may be difficult. Overlapping σ-factor binding sites were detected frequently in *Escherichia coli*: e.g. 38 genes were assigned to 4 different sigma factors and 2 genes were even assigned to 6 sigma factors (Cho et al. 2014).

In this study, we used an in vitro transcription system and in vivo methods (overexpression of sigma genes to drive transcription from the promoters transcriptionally fused to the *gfp*uv reporter, use of *sig*-deletion strains) to reliably determine which sigma factors control transcription driven by individual tested promoters in *C. glutamicum*. We analyzed both housekeeping σ factors and ECF σ factors involved in stress responses. Overexpression of the *sig* genes encoding ECF σ factors usually results in the stronger expression of the σ factor-specific genes, even in the absence of the respective stress signal. This is advantageous particularly when the conditions under which the respective sigma factor is active are not known. The consistency of results achieved by the in vivo and in vitro techniques provided reliable promoter classification as well as new data on the analyzed promoters.

## Materials and methods

### Bacterial strains, plasmids, oligonucleotide primers and growth conditions

The bacterial strains and plasmids used are listed in Table 1. The oligonucleotide primers are listed in Additional file 1: Table S1. *E. coli* DH5α was used for cloning purposes. Wild-type (WT) *C. glutamicum* ATCC 13032 and its deletion derivatives *C. glutamicum* Δ*sigB*, ∆*sigE*, Δ*sigH* and Δ*sigM* were used as hosts for testing the activities of promoters cloned in the promoter-test vector pEPR1. *E*. *coli* was cultivated aerobically in 500-ml flasks containing 80 ml of 2xYT medium (Sambrook and Russel 2001) on a rotary shaker at 150 rpm and 37 °C. *C. glutamicum* was cultivated in 500-ml flasks with 80 ml of complete 2xYT medium (Sambrook and Russel 2001) or in minimal CGXII medium (Keilhauer et al. 1993) with protocatechuic acid at a concentration of 0.03 g/l on a rotary shaker at 150 rpm and 30 °C. Kanamycin (30 μg/ml), tetracycline (10 μg/ml), spectinomycin (200 μg/ml) or ampicillin (100 μg/ml) was added to the selective media when appropriate.

**Table 1:** Strains and plasmids used in this study

Strains	Relevant characteristics	Source/reference/application
*E. coli*		
DH5α	Cloning host	Hanahan (1985)
*C. glutamicum*		
WT	ATCC 13032, wild type	ATCC
∆*sigB*	Deletion in *sigB*	Larisch et al. (2007)
∆*sigE*	Deletion in *sigE*	Park et al. (2008)
*∆sigH*	Deletion in *sigH*	Zemanová et al. (2008)
∆*sigM*	Deletion in *sigM*	Nakunst et al. (2007)
Plasmids		
pEC-XT99A	*E. coli*–*C. glutamicum* expression vector, Tc^R^, IPTG-inducible *trc* promoter	Kirchner and Tauch (2003)
pEKEx3	*E. coli*–*C. glutamicum* expression vector, Sp^R^, IPTG-inducible *tac* promoter	Hoffelder et al. (2010)
pEPR1	*E. coli*–*C. glutamicum* promoter-test vector, Km^R^, promoterless *gfp*uv as a reporter	Knoppová et al. (2007)
pEPR-P2*sigA*	pEPR1 with P2*sigA*	This work
pEPR-P*fba*	pEPR1 with P*fba*	This work
pEPR-P*cg2556*	pEPR1 with P*cg2556*	This work
pEPR-P*cmt1*	pEPR1 with P*cmt1*	This work
pEPR-P*sigB*	pEPR1 with P*sigB*	This work
pEPR-P*rshA*	pEPR1 with P*rshA*	This work
pEPR-P*trxB1*	pEPR1 with P*trxB1*	This work
pRLG770	*E. coli* vector for in vitro transcription, *rrnB* terminator, Ap^R^	Ross et al. (1990)
pRLG770P2*sigA*	pRLG770 with P2*sigA*	This work
pRLG770P*fba*	pRLG770 with P*fba*	This work
pRLG770P*cg2556*	pRLG770 with P*cg2556*	This work
pRLG770P*cmt1*	pRLG770 with P*cmt1*	This work
pRLG770 P*sigB*	pRLG770 with P*sigB*	This work
pRLG770P*rshA*	pRLG770 with P*rshA*	This work
pRLG770P*trxB1*	pRLG770 with P*trxB1*	This work

*IPTG* isopropyl-β-thiogalactopyranoside, *Tc* ^*R*^ tetracycline resistance, *Sp* ^*R*^ spectinomycin resistance, *Km* ^*R*^ kanamycin resistance, *Ap* ^*R*^ ampicillin resistance

### DNA manipulations

DNA isolation, PCR, transformation of *E. coli*, DNA cloning and DNA analysis were performed using standard methods (Sambrook and Russel 2001). Genomic DNA from *C. glutamicum* was isolated as described (Eikmanns et al. 1994). *C. glutamicum* cells were transformed by electroporation (van der Rest et al. 1999).

### Construction of the two-plasmid system for assignment of sigma factors to promoters in vivo

We constructed a system for the in vivo identification of *C. glutamicum* promoters recognized by RNAP containing a particular sigma factor, which is based on the two-plasmid *C. glutamicum* strains similar to that developed for the identification of σ^E^-dependent promoters in *E. coli* (Rezuchova and Kormanec 2001). Promoters carried on the *Bam*HI–*Pst*I DNA fragments (amplified using PCR, oligonucleotide primers listed in Additional file 1: Table S1 and *C. glutamicum* chromosome as a template) were cloned in the promoter-test vector pEPR1 containing the promoterless *gfp*uv reporter gene (Knoppová et al. 2007). The genes encoding seven different sigma factors were cloned under the P*trc* promoter inducible with isopropyl-β-d-1-thiogalactopyranoside (IPTG) in the expression vector pEC-XT99A (Kirchner and Tauch 2003). The sequences of all inserts were checked by sequencing. Analogous constructs carrying the *sig* genes under P*tac* in the expression vector pEKEx3 (Hoffelder et al. 2010) were kindly supplied by Taniguchi and Wendisch (2015). There was negligible gene expression from pEC-XT99A without IPTG when we tested the model expression of the *gfp*uv gene, whereas the expression of the *gfp*uv gene from pEKEx3 without IPTG addition was 20–30% of the maximum after IPTG induction (data not shown). *C. glutamicum* strains harboring both the pEPR1 + promoter and pEC-XT99A (or pEKEx3) + *sig* gene were obtained by successive transformation. The presence of correct plasmids in two-plasmid strains was checked by restriction enzyme analysis and PCR.

### In vivo promoter activity measurements

To evaluate the effect of *sig* gene overexpression on the activity of a particular promoter, the assay was performed as follows: The two-plasmid strain carrying the *sig* gene in the expression vector and a promoter in the promoter test vector pEPR1 was cultivated aerobically in 80 ml 2xYT medium at 30 °C. IPTG (1 mM) was added to the culture when OD_600_ reached 1 to overexpress a particular *sig* gene. Samples of the culture were then taken at various time points (mostly 0, 3, 6 and 24 h). The cells were washed with phosphate-buffered saline pH 8.0 (PBS) (Sambrook and Russel 2001) with 1 mM phenylmethylsulfonyl fluoride and 2 mM 2-mercaptoethanol, concentrated in 0.5 ml of cell suspension to a final OD_600_ = 24 in PBS and disrupted with a FastPrep homogenizer (MP Biomedical) (3 × 60 s at speed 6 m/s with Lysing Matrix B in 2-ml tubes). After centrifugation (20 min at 15,000×*g*) the fluorescence of the cell-free extract was measured with a Saphire2 microplate spectrophotometer (Tecan; excitation wavelength, 397 nm; emission wavelength 509 nm). Protein concentration in the extract was determined by Bradford assay and promoter activity was expressed in arbitrary units/mg protein. Cells harboring the pEPR1 construct and the expression vector without a *sig* gene were used as a control.

### In vitro transcription assay

The multiple-round in vitro transcription assay was in principle performed using the recently described system (Holátko et al. 2012). Both the RNAP core and sigma factors from *C. glutamicum* were isolated as described previously, using the *C. glutamicum rpoC*-H8 strain producing the RNAP core and *E. coli* BL21 (DE3) with pET-22b(+) constructs producing individual *C. glutamicum* σ factors (Holátko et al. 2012; Šilar et al. 2016). The reconstituted holo-RNAP was prepared by mixing the RNAP core (100 nM) and the respective sigma factor in a molar ratio of 1:30 and incubating for 10 min at 37 °C. The transcription mixture contained ATP, CTP and GTP (200 µM each), 10 µM UTP and 3 µM α^32^P-UTP. The reaction was run for 10 min at 37 °C. All promoter fragments (approx. 70 nt) were cloned in the vector pRLG770 (using *Eco*RI and *Hin*dIII sites) in such a way that a 150-nt transcript (terminated at the *rrnB* terminator) was produced by the in vitro transcription (Holátko et al. 2012). The produced radiolabeled transcripts were separated by electrophoresis on 5.5% polyacrylamide gels (PAGE) with 7 M urea. The transcripts in dried gels were detected by exposure to phosphorimaging screen (6–24 h), followed by scanning with a Molecular Imager FX (BIO-RAD). Electrophoresis gel data from the imaging systems were visualized and analyzed with *Quantity One* 1-D analysis software. All assays were performed at least three times and consistent results were obtained. Representative results are shown.

### Tryptic digestion of in-gel proteins and liquid chromatography-tandem mass spectrometry (LC–MS/MS) analysis

The proteins extracted from the *C. glutamicum* cells disrupted by sonication were run in SDS-PAGE gels. The gel slice containing a bend corresponding to 25 kDa protein was digested with trypsin (100 ng; Promega) overnight in a cleavage buffer containing 25 mM 4-ethylmorpholine acetate, and the resulting peptides were subjected to an LC–MS/MS analysis using collision-induced fragmentation in a Synapt G2Si mass spectrometer (Waters) coupled to an ACQUITY UPLC M-class system (HSS T3 1.8 μm column, 75 μm × 150 mm). For protein identification, the tandem mass spectra were searched against the NCBI bacterial database using an in-house Mascot search engine.

## Results

### σ^H^-dependent promoters: P*rshA* and P*trxB1*

**Fig. 1:**
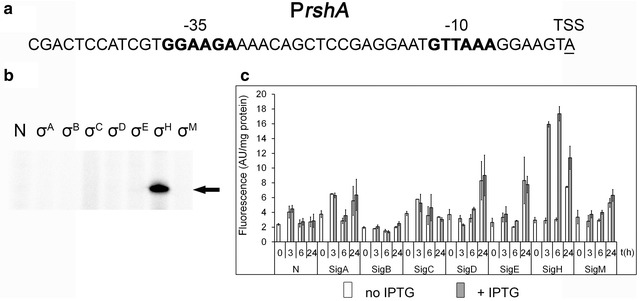
Assignment of a sigma factor to the P*rshA* promoter. **a** Sequence of the *rshA* (*cg0876*) promoter (Pfeiffer-Sancar et al. 2013). The −35 and −10 sequence elements are in *bold*, the transcriptional start site is *underlined*. **b** In vitro transcription with P*rshA* as a template. Individual sigma factors associated with RNAP are shown at the *top*. *N* no sigma factor protein was added to the RNAP core. The specific transcript is indicated with an *arrow*. **c** P*rshA* activity determined with the two-plasmid system in *C. glutamicum* ∆*sigH* by measuring the fluorescence intensity of the Gfpuv reporter. The sigma factors whose genes were overexpressed are indicated at the *bottom*. IPTG was added at time 0. *AU* arbitrary units. The standard deviations (SDs) of three measurements are depicted as *error bars*

σ^H^ is the most studied ECF sigma factor, which forms a transcriptional regulatory network enabling the *C. glutamicum* cell to respond to temperature, oxidative and growth-phase induced stresses (Busche et al. 2012; Ehira et al. 2009; Toyoda and Inui 2016b; Toyoda et al. 2015). Due to its role in the expression of the genes encoding regulatory proteins and prominent position in the regulatory network, it is a likely candidate for a global regulator in *C. glutamicum* (Schröder and Tauch 2010). The *rshA* gene that encodes the anti-σ^H^ factor was localized immediately downstream of the *sigH* gene and forms an operon with *sigH* (Busche et al. 2012). The *rshA* gene is transcribed from P*rshA* (internal to *sigH*) in addition to the P*sigH* promoters found upstream of the operon (Busche et al. 2012). P*rshA* sequence elements −35 GGAAGA and −10 GTTAAA (Fig. 1a) conform to the consensus of σ^H^-dependent promoters, however the expression of *rshA* was not found to be up-regulated in *C. glutamicum* by the microarray analysis (Ehira et al. 2009). In vitro transcription assays resulted in a single specific band produced by RNAP with σ^H^ (Fig. 1b). The in vivo analysis with pEC-XT99A and pEPR1 carrying the *sig* genes and P*rshA*, respectively, in the *C. glutamicum* ∆*sigH* strain unequivocally demonstrated that P*rshA* is controlled by σ^H^ (Fig. 1c). Very similar results were obtained when the *C. glutamicum* WT was used (data not shown).

**Fig. 2:**
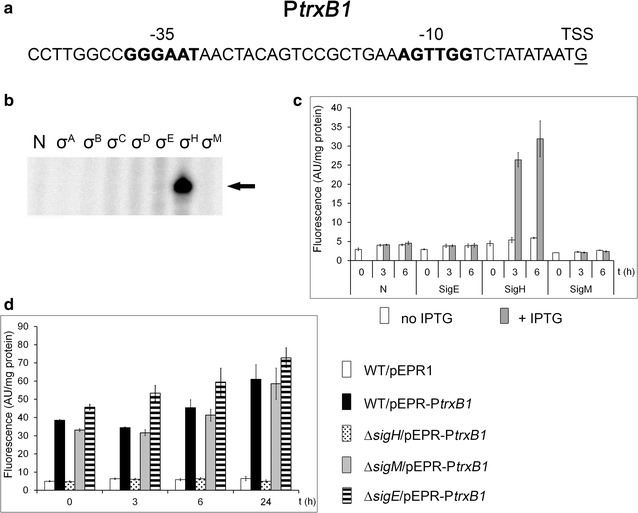
Assignment of a sigma factor to the P*trxB1* promoter. **a** Sequence of the P*trxB1* promoter (Nakunst et al. 2007). The −35 and −10 sequence elements are in *bold*, the transcriptional start site is *underlined*. **b** In vitro transcription with P*trxB1* as a template. Individual sigma factors associated with RNAP are shown at the *top*. *N* no sigma factor was added to the RNAP core. The specific transcript is indicated with an *arrow*. **c** P*trxB1* activity determined with the two-plasmid system in *C. glutamicum* ∆*sigH* by measuring the fluorescence intensity of the Gfpuv reporter. The sigma factors whose genes were overexpressed are indicated at the *bottom*. IPTG was added at time 0. **d** P*trxB1* activity in *C. glutamicum* ∆*sigH*, ∆*sigM* or ∆*sigE* strains carrying a single plasmid, pEPR-P*trxB1*. The values for the WT strain carrying the empty vector pEPR1 are shown as a control. *AU* arbitrary units. The SDs of three measurements are depicted as *error bars*

The promoter of the *trxB1* gene was first described as σ^M^-dependent (Nakunst et al. 2007) but as σ^H^-dependent in another study (Busche et al. 2012). The *trxB1* gene encodes a disulfide oxido-reductase that catalyzes a wide spectrum of redox reactions in the cell and is involved in oxidative stress response. A transcriptional start site of *trxB1* was determined by RACE (Nakunst et al. 2007) and later confirmed by RNA sequencing (Pfeifer-Sancar et al. 2013). A single promoter of the gene with the key promoter elements −35 GGAATA and −10 GTTGGT (Fig. 2a) was thus localized. The core conserved sequence motifs −35 GGAA and −10 GTT match the proposed consensus sequences of both σ^M^-specific (Nakunst et al. 2007) and σ^H^-specific (Busche et al. 2012) promoters. The in vitro transcription assay showed that only σ^H^ recognized P*trxB1* (Fig. 2b).

In vivo analysis using the two-plasmid system in the *C. glutamicum* ∆*sigH* strain also clearly indicated that it was only the overexpression of σ^H^ that triggered a sharp increase in the P*trxB1* activity (Fig. 2c). Very similar results were obtained when *C. glutamicum* WT was used (data not shown). To detect whether some other stress sigma factor at least weakly contributes to P*trxB1* activity in vivo, the effects of deletions in the genes *sigH*, *sigM* and *sigE* on the P*trxB1* activity during growth were tested. As shown in Fig. 2d, the reporter fluorescence measured with strains carrying only pEPR1-P*trxB1* remained at the level of the control (WT) in the ∆*sigM* and ∆*sigE* strains, whereas it was approximately 14-fold lower in the ∆*sigH* strain and essentially at the same level as that obtained with the strain only carrying the empty vector pEPR1. We therefore concluded that the P*trxB1* promoter is σ^H^-specific.

### A σ^E^- and σ^H^-dependent promoter: P*sigB*

**Fig. 3:**
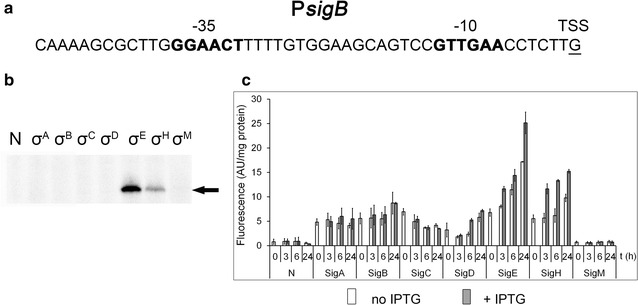
Assignment of sigma factors to the P*sigB* promoter. **a** Sequence of the P*sigB* promoter (Halgasova et al. 2001). The assumed −35 and −10 sequence elements are in *bold*, the transcriptional start site is *underlined*. **b** In vitro transcription with P*sigB* as a template. Individual sigma factors associated with RNAP are shown at the *top*. *N* no sigma factor protein was added to the RNAP core. The specific transcripts are indicated with an *arrow*. **c** P*sigB* promoter activity determined using the two-plasmid *C. glutamicum* ∆*sigH* by measuring the fluorescence intensity of the Gfpuv reporter. The sigma factors whose genes were overexpressed are indicated at the *bottom*. IPTG was added at time 0. *AU* arbitrary units. The SDs of three measurements are depicted as *error bars*

Next, we tested the effect of the *sigE* gene deletion on the transcriptional activity of the *cg1266* gene. This gene was suggested to be σ^E^-dependent (Park et al. 2008) but we did not detect any potential σ^E^-dependent promoter (data not shown). Based on the similarity of the promoter region of the *C. glutamicum sigB* gene to the *Mycobacterium tuberculosis sigB* promoter (controlled by σ^E^ and σ^H^) it was suggested that the *C. glutamicum sigB* may be under the control of σ^E^ (Halgasova et al. 2002). The *sigB* gene exhibited a higher level of transcription when *sigH* was overexpressed and was therefore considered to be σ^H^-dependent (Ehira et al. 2009). The P*sigB* sequence elements −35 GGAA and −10 GTT conform to the consensus of σ^H^-dependent promoters, however, the σ^H^ dependence was not proved by microarrays when ∆*sigH* (Ehira et al. 2009) or ∆*rshA* strains (Busche et al. 2012) were used. This gave us a hint that still another sigma factor is involved in *sigB* transcription. We carried out the in vitro transcription assays with all seven *C. glutamicum* sigma factors. Bands were detected when RNAP + σ^E^ or σ^H^ were applied (Fig. 3b). Specific band intensities based on three in vitro assays were quantified using *Quantity One* 1-D software, which showed that approximately 5.5-fold more transcript was produced with RNAP + σ^E^ than with RNAP + σ^H^. The results of in vivo analysis using the two-plasmid system in the *C. glutamicum* WT suggested that the transcription from P*sigB* is induced by the overexpression of σ^H^ and significantly less by the overexpression of σ^E^ (data not shown). To test whether RNAP + σ^E^ can initiate transcription from P*sigB* in vivo more efficiently when σ^H^ does not compete with σ^E^, we used the two-plasmid assay in the ∆*sigH* strain with all seven sigma factors. As shown in Fig. 3c, the overexpression of both σ^H^ and σ^E^ increased expression of the *gfp*uv reporter gene from P*sigB*. These results clearly demonstrated that RNAP + σ^E^ can drive transcription from P*sigB* but σ^H^ competes with σ^E^ for RNAP or binding to the promoter.

### A σ^C^-dependent promoter: P*cg2556*

**Fig. 4:**
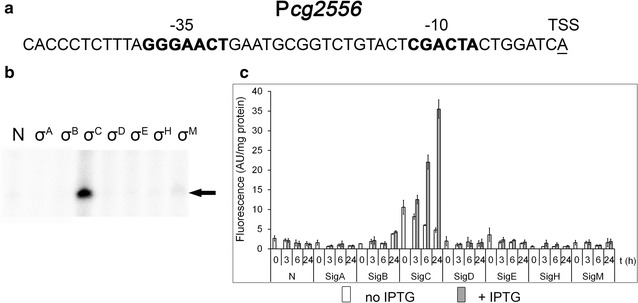
Assignment of a sigma factor to the P*cg2556* promoter. **a** Sequence of the *cg2556* (*cgR_2208*) promoter (Toyoda and Inui 2016a). The −35 and −10 sequence elements are in *bold*, the transcriptional start site is *underlined*. **b** In vitro transcription with P*cg2556* as a template. Individual sigma factors associated with RNAP are shown at the *top*. *N* no sigma was added to the RNAP core. The specific transcript is indicated with an *arrow*. **c** P*cg2556* activity determined with the two-plasmid system in *C. glutamicum* WT by measuring the fluorescence intensity of the Gfpuv reporter. The sigma factors whose genes were overexpressed are indicated at the *bottom*. The SDs of three measurements are depicted as *error bars*

Expression of the genes regulated by σ^C^ has recently been described (Toyoda and Inui 2016a). We selected the promoter of the *C. glutamicum* ATCC 13032 *cg2556* gene encoding an uncharacterized iron-regulated membrane protein (corresponding to the cgR_2208 gene in the sequence of *C. glutamicum* R; Toyoda and Inui 2016a) to test sigma dependency of its promoter. In vitro transcription assays confirmed that *cg2556* transcription is σ^C^-specific (Fig. 4b). The in vivo two-plasmid assay produced the same result (Fig. 4c).

### A σ^D^-dependent promoter: P*cmt1*

**Fig. 5:**
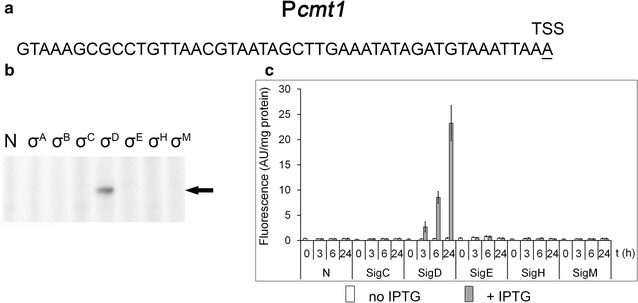
Assignment of a sigma factor to the P*cmt1* promoter. **a** Sequence of the *cmt1* promoter region. **b** In vitro transcription with P*cmt1* as a template. Individual sigma factors associated with RNAP are shown at the *top*. *N* no sigma factor was added to the RNAP core. The specific transcript is indicated with an *arrow*. **c** P*cmt1* promoter activity determined with the two-plasmid system in *C. glutamicum* WT by measuring the fluorescence intensity of the Gfpuv reporter. The sigma factors whose genes were overexpressed are indicated at the *bottom*. IPTG was added at time 0. *AU* arbitrary units. The SDs of three measurements are depicted as *error bars*

RNA sequencing studies suggested that there is a group of genes which are σ^D^-dependent (Busche and Kalinowski, unpublished). We selected the promoter of *cmt1* (encoding trehalose corynomycolyl transferase) to test its σ^D^ dependency. In vitro transcription assays only provided a specific band when RNAP + σ^D^ was used (Fig. 5b). The in vivo two-plasmid analyses in the WT strain using all ECF σ factors confirmed that P*cmt1* is σ^D^-dependent (Fig. 5c). To date no consensus sequence of the promoters recognized by RNAP + σ^D^ has been proposed. The involvement of the *cmt1* gene and other potentially σ^D^-dependent genes in stress response is currently being studied.

### The quest for a σ^M^-dependent promoter

The sigma factor σ^M^ was found to be involved in oxidative stress response in *C. glutamicum*. The σ^M^-dependent transcription of 23 genes was suggested by using microarray analyses of the *C. glutamicum* WT and ∆*sigM* strain (Nakunst et al. 2007). Promoter sequences of four of these genes (P*trxB*, P*trxC*, P*trxB1* and P*sufR*) were localized by determination of the respective transcriptional start sites (TSSs). Three of the genes (*trxB*, *trxC* and *sufR*) were found to be σ^H^-dependent by Ehira et al. (2009). We analyzed all these four promoters using both in vivo and in vitro techniques. In all cases only σ^H^-dependent transcription was detected (data not shown). We were thus unable to confirm the σ^M^ dependency of any of these promoters. The failure to detect an in vitro transcript with RNAP + σ^M^ might be due to the limitations of the technique used. In contrast to all other σ factors purified for the in vitro assays, most of the σ^M^ protein was detected in the insoluble fraction after its isolation from *E. coli* extracts using affinity chromatography (data not shown). Various modifications of the protocol did not improve the ratio of the soluble/insoluble fraction. The σ^M^ protein was therefore denatured and renatured. Since no σ^M^-specific promoter was detected by the in vitro assay, there is a possibility that the renatured σ^M^ was not functional in vitro. Another reason for failing to prove σ^M^-dependent transcription using the in vitro transcription may be missing activators. To test whether σ^M^ is present in the *C. glutamicum* ∆*sigM* cells carrying the pEC-XT99A vector with cloned *sigM* after IPTG induction in the in vivo assay, we analyzed the proteins of the cell extract by LC–MS/MS. Among other proteins the presence of the σ^M^ protein was confirmed by the identification of four peptides which covered 22% of the σ^M^ protein sequence (Additional file 1: Table S2). Thus, the failure to demonstrate σ^M^ dependency for any of these promoters in vivo was not due to the absence of σ^M^ expression. The elucidation of σ^M^ function and finding σ^M^-controlled promoters needs further investigation.

### A σ^A^-dependent promoter: P2*sigA*

**Fig. 6:**
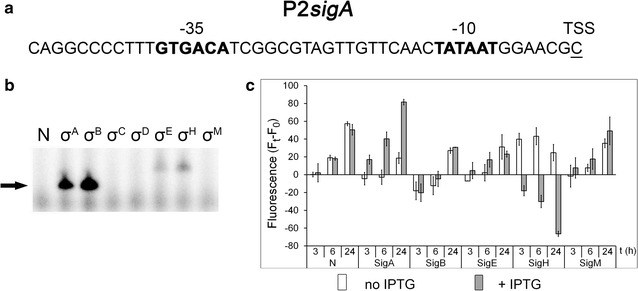
Assignment of sigma factors to the P2*sigA* promoter. **a** Sequence of the P2*sigA* promoter (Halgasova et al. 2001). **b** In vitro transcription with P2*sigA* as a template. Individual sigma factors associated with RNAP are shown at the *top*. The specific transcripts are indicated with an *arrow*. **c** P2*sigA* promoter activity determined with the two-plasmid system in *C. glutamicum* WT by measuring the fluorescence intensity of the Gfpuv reporter. F_t_ − F_0_ is the difference between the fluorescence at the sampling time (3, 6, 24 h) and the fluorescence at time 0 (before sigma gene induction). The sigma factors whose genes were overexpressed are indicated at the *bottom*. The SDs of three measurements are depicted as *error bars*

It is difficult to reliably prove that a promoter is σ^A^-specific in *C. glutamicum* since σ^A^ is present in the cell during all growth phases and under most conditions, and deletion of the *sigA* gene would be lethal. The promoters are usually considered “vegetative” or “housekeeping” if their −35 and −10 promoter element sequences match the generally accepted consensus of housekeeping promoters and the respective genes are expressed during exponential growth under optimal conditions. We selected the P2*sigA* promoter of the *sigA* gene encoding the primary sigma factor to test whether the designed methods can prove its assumed σ^A^ dependency. P2*sigA* was localized by the determination of *sigA* TSS (Halgasova et al. 2001). According to the transcriptional profile of the *sigA* gene (expressed mainly in the exponential growth phase; Larisch et al. 2007) it is supposed to be σ^A^-dependent. The sequences of the key promoter elements, −35 GTGACA and −10 TATAAT (Fig. 6a), are closely similar to the defined consensus sequence of the σ^A^-dependent promoters in *C. glutamicum* (Pátek and Nešvera 2011; Pfeifer-Sancar et al. 2013) and σ^70^-dependent promoters in *E. coli* (Lisser and Margalit 1993). The in vitro transcription assays showed that RNAP + σ^A^, but also RNAP + σ^B^ provide specific transcripts with the P2*sigA* promoter as a template (Fig. 6b). The values of fluorescence obtained from the two-plasmid system in *C. glutamicum* WT using pECXT-99A with *sigA* or *sigB* were lower than those obtained with other sigma factors and the control (empty pECXT-99A) as well. We also observed this phenomenon for other σ^A^- and σ^B^-dependent promoters (data not shown). To see the effect of σ factor gene overexpression more clearly, the fluorescence intensity values were expressed as differences between the fluorescence at the sampling time (3, 6, 24 h) and the fluorescence at time 0 (Fig. 6c). Only the overexpression of *sigA* (from pEC-XT99A) resulted in an increase in P2*sigA* promoter activity in both the exponential and stationary phase, whereas the overexpression of other sigma factors did not change its activity, or even decreased it (σ^H^) (Fig. 6c). This effect may be due to the competition of σ^H^ with σ^A^ for RNAP. Similarly, the low activity of P2sigA when σ^B^ was overexpressed could be the consequence of the competition of σ^A^ with σ^B^ for RNAP or binding the respective holoenzyme (RNAP + σ^A^ or RNAP + σ^B^) to the promoter. We conclude that P2sigA is predominantly σ^A^-dependent and may probably also be active with σ^B^ in vivo under specific conditions (e.g. stress and the stationary phase).

### A σ^B^- dependent promoter: P*fba*

**Fig. 7:**
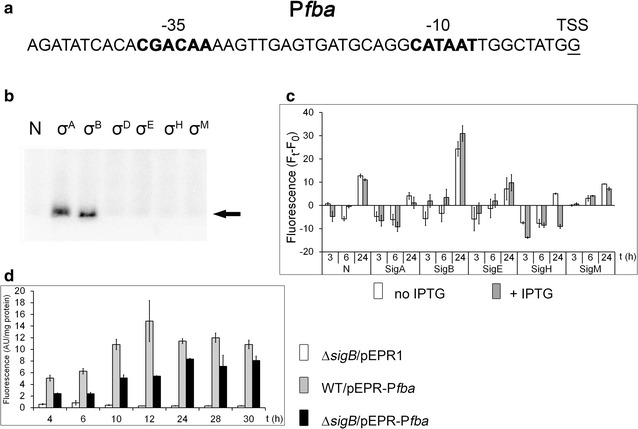
Assignment of sigma factors to the P*fba* promoter. **a** Sequence of the P*fba* promoter (Ehira et al. 2008). **b** In vitro transcription with P*fba* as a template. Individual sigma factors associated with RNAP are shown at the *top*. The specific transcripts are indicated with an *arrow*. **c** P*fba* promoter activity determined with the two-plasmid system in *C. glutamicum* WT (with the *sig*-constructs in the expression vector pEKEx3) by measuring the fluorescence intensity of the Gfpuv reporter. F_t_ − F_0_ is the difference between the fluorescence at the sampling time (3, 6, 24 h) and the fluorescence at time 0 (before sigma gene induction). The sigma factors whose genes were overexpressed are indicated at the *bottom*. IPTG was added at time 0. **d** Activity of P*fba* in *C. glutamicum* WT and Δ*sigB*. *C. glutamicum* Δ*sigB* with empty vector pEPR1 served as a control. The SDs of three measurements are depicted as *error bars*

The *fba* gene (encoding fructose 1,6-bisphosphate aldolase) was found to be downregulated in the *sigB* deletion strain both under conditions of oxygen deprivation and during aerobic cultivation (Ehira et al. 2008). The gene is involved in glucose metabolism and is mostly expressed during exponential growth whereas its expression decreases in the transition phase. The sequences of the key promoter elements (Ehira et al. 2008), −35 CGACAA and −10 CATAAT (Fig. 7a) are very similar to those of the proposed consensus of σ^A^-specific promoters (Pátek and Nešvera 2011; Pfeifer-Sancar et al. 2013). In vitro transcription assays showed that both σ^A^ and σ^B^ with RNAP produce specific signals with P*fba* (Fig. 7b). In vivo analysis using the two-plasmid system in the *C. glutamicum* WT strain with the expression vector pEC-XT99A carrying cloned *sig* genes did not provide an increase in promoter activity with any σ factor (data not shown). However, an alternative system utilizing the expression vector pEKEx3 with cloned *sig* genes and pEPR1-P*fba* proved that P*fba* is recognized by σ^B^ under the conditions used (Fig. 7c). A slight increase was also observed with σ^E^. Since we found that the promoter of the *sigB* gene is transcribed by RNAP + σ^E^, this effect may be explained by the indirect effect of *sigE* overexpression. Activity of P*fba* was further tested in the single-plasmid strains *C. glutamicum* WT and ∆*sigB* carrying pEPR1-P*fba*. The activity of P*fba* was significantly lower in the ∆*sigB* strain than in WT, but was still higher than the activity exhibited by the ∆*sigB* strain carrying an empty vector pEPR1 (Fig. 7d). The observed substantial residual activity of P*fba* in *C. glutamicum* Δ*sigB* is in agreement with the recognition of this promoter by σ^A^ proved in vitro. These results suggest that transcription from P*fba* is mainly driven by RNAP + σ^B^ even during exponential growth, and σ^A^ may substitute for σ^B^ under some specific conditions.

## Discussion

Expression of sigma factor genes in bacteria is organized into cascades or networks (Qiu et al. 2013; Cho et al. 2014). Therefore, it is sometimes difficult to distinguish between the direct and indirect effects of the overexpression or deletion of *sig* genes in studies of the dependence of promoters on sigma factors. The genome-level in vivo approaches to analyzing σ-specific regulons using microarrays or ChIP-chip techniques may therefore be negatively affected by these regulatory interactions.

Thus the results of in vivo techniques may be overshadowed by secondary effects caused by the cascade or network nature of σ regulation, the competition of σ factors for RNAP and promoters, or the activities of transcriptional regulators. In contrast, the in vitro reaction, in which the DNA template is transcribed from a single promoter by a purified RNAP core with a single σ factor, can avoid such interactions. An in vitro transcription system which mimics many features of in vivo transcription thus provides results that are free of indirect effects. On the other hand, the in vitro transcription may produce some artifacts, since some promoters may require activators or appropriate DNA conformation (superhelicity) or other type of physiological control for their natural activity.

To compensate for the drawbacks of each of these approaches, both in vivo and in vitro methods should be applied for the analysis of sigma factor–promoter interactions. By combining the results of in vitro and in vivo experiments, one can achieve an unambiguous assignment of sigma factors to promoters.

The main aim of this work was to integrate the results of our newly developed in vitro and in vivo techniques so that we can reliably classify individual promoters according to their σ factor dependency. This approach proved to be useful, and we were able to find a representative example of a promoter for every sigma factor with the exception of σ^M^. The achieved results show that the system can produce data that is almost free of secondary effects. We were also able to convincingly document the recognition overlap of two σ factors at a single promoter (σ^A^/σ^B^, σ^E^/σ^H^).

The *rshA* gene, which is located immediately downstream of *sigH*, encodes the anti-σ^H^ factor. The gene is transcribed together with the *sigH* from σ^A^-dependent promoters and separately as a monocistronic *rshA* transcript from P*rshA* which was proposed to be σ^H^-dependent (Busche et al. 2012). This arrangement probably ensures the rapid shutdown of the σ^H^-dependent stress response as soon as the stress conditions are over. We have now confirmed by both in vivo and in vitro techniques that P*rshA* is a σ^H^-specific promoter (Fig. 1). Similarly, P*trxB1* was shown to be a σ^H^-specific promoter (Fig. 2). Neither the in vitro nor in vivo assay generated a signal with any other sigma factor. The P*trxB1* promoter activity measurements using the ∆*sigH*, ∆*sigM* and ∆*sigE* strains showed that *sigH* deletion completely eliminated its activity, whereas the *sigM* and *sigE* deletions did not change or even increased its activity (Fig. 2d). The activities of P*trxB*, P*trxC* and P*sufR* that were also predicted to be σ^M^-dependent in a study based on a *sigM* deletion strain (Nakunst et al. 2007) but σ^H^-dependent according to the disruption or overexpression of *sigH* (Ehira et al. 2009) were completely eliminated in ∆*sigH* as well (data not shown). The in vivo two-plasmid system also showed that P*trxB*, P*sufR* and P*trxC* are σ^H^-specific, although the presence of σ^M^ in the *C. glutamicum* cells after the induction of *sigM* expression was proved by mass spectrometry.

We have recently shown that the promoters P1*clgR*, P2*dnaK* and P2*dnaJ2* are recognized by both σ^E^ and σ^H^ (Šilar et al. 2016). To date, no exclusively σ^E^-specific *C. glutamicum* promoter has been reported. P*sigB* was also found to be controlled by σ^E^ and σ^H^ in this study, just like the *M. tuberculosis sigB* promoter (Rodrigue et al. 2006). This is in agreement with suggestions that *C. glutamicum* σ^B^ plays a role as a general stress response σ factor and as a back-up housekeeping σ for stress conditions (Halgasova et al. 2002; Larisch et al. 2007).

The σ^C^-dependent promoters seem to be very specific and are probably recognized exclusively by σ^C^, although their consensus sequence elements −35 GGGAACT and −10 CGACTA (Toyoda and Inui 2016a) contain the same −35 tetramer GGAA as σ^H^-dependent promoters. Both in vitro and in vivo methods clearly confirmed the assignment of σ^C^ to P*cg2556*. Similarly, the two methods coincidentally indicated that the P*cmt1* promoter is σ^D^-specific. The consensus sequence of σ^D^-specific promoters is currently being explored (Busche and Kalinowski, unpublished data).

In contrast to stress promoters, σ^A^- and σ^B^-dependent promoters exhibited relatively high expression in all growth phases even without the overexpression of a *sig* gene. The cell levels of σ^A^ and σ^B^ are apparently high enough to drive expression from the tested promoters. Moreover, as was shown for the σ^B^-dependent promoters P*pqo* (Šilar et al. 2016) and P*fba*, σ^A^ is able to partially substitute for missing σ^B^ in ∆*sigB* strain. The ability of σ^A^ and σ^B^ to recognize P*fba* was confirmed by the in vitro transcription assays. This interchangeability of σ^A^ and σ^B^ was also shown for the typical housekeeping promoters P*per* (Šilar et al. 2016) and P2*sigA* (Fig. 6b). This is not surprising since the amino acid sequences of σ^A^ and σ^B^ in protein regions 2.4 and 4.2 which recognize the −10 and −35 promoter motifs respectively are highly similar, and promoter consensus sequences of σ^A^ and σ^B^ could not be distinguished. These findings for *C. glutamicum* are analogous to those for *E. coli* σ^70^ and σ^S^ (Typas et al. 2007).

Detailed knowledge of functions of σ factors is a prerequisite for their engineering aimed at modulation of transcriptional regulatory network and, consequently, strain improvement for biotechnological purposes (Tripathi et al. 2014). Mutagenesis of σ factors or other transcriptional regulators and screening the mutants for their ability to reprogram cellular metabolism and regulation to the desired phenotype is a basis of newly developed global transcriptional machinery engineering method (for a review, see Tyo et al. 2007; Lanza and Alper 2011; Liu and Jiang 2015). For the first time, this approach was used for improvement of ethanol tolerance and production in *Saccharomyces cerevisiae* by mutagenesis of transcription factor Spt15p (Alper et al. 2006). Random mutagenesis of *E. coli* primary σ^70^ factor was found to result in global perturbations of the transcriptome and the mutants exhibiting ethanol tolerance, increased lycopene production and multiple tolerance phenotypes, respectively, were obtained (Alper and Stephanopoulos 2007). Screening the library of *E. coli* σ^70^ factor-mutants under cyclohexane pressure resulted in obtaining the strains highly tolerant to this solvent (Zhang et al. 2015). *E. coli* strains accumulating hyaluronic acid effectively were obtained also by screening the σ^S^-mutants (Yu et al. 2008).

The use of two alternative vectors (pEC-XT99A and pEKEx3) for overexpressing *sig* genes gave essentially the same results, which widens the choice for in vivo promoter analysis. A comparison of the results obtained with both in vivo and in vitro approaches proved to be useful for the unequivocal assignment of a sigma factor to a single promoter. Combining the advantages of in vivo and in vitro techniques can minimize the drawbacks of the techniques as stand-alone approaches and finally provide reliable sigma factor–promoter assignment.

## Additional file

**Additional file 1.** Additional tables.

## Abbreviations

ECF: extracytoplasmic function
IPTG: isopropyl-β-d-1-thiogalactopyranoside
LC–MS/MS: liquid chromatography-tandem mass spectrometry
OD_600_: optical density at 600 nm
PBS: phosphate-buffered saline
RNAP: RNA polymerase
SD: standard deviation
SDS-PAGE: sodium dodecylsulfate polyacrylamide gel electrophoresis
TSS: transcriptional start site
WT: wild-type
