# Supplementary material for: Assignment of sigma factors of RNA polymerase to promoters in Corynebacterium glutamicum
Source: AMB Express. 2017 Jun 24;7:133. doi: 10.1186/s13568-017-0436-8 (PMC5483222; doi:10.1186/s13568-017-0436-8)
Supplement: Supplementary file 1 — Additional file 1. Additional tables. [file 13568_2017_436_MOESM1_ESM.pdf]

Assignment of sigma factors of RNA polymerase to promoters in *Corynebacterium glutamicum*

Hana Dostálová[1], Jiří Holátko[1], Tobias Busche[2], Lenka Rucká[1], Andrey Rapoport[1], Petr Halada[1], Jan Nešvera[1], Jörn Kalinowski[2] and Miroslav Pátek[1]*

[1]*Institute of Microbiology of the CAS, v. v. i., Vídeňská 1083, 14220 Prague 4, Czech Republic*
[2]*Center for Biotechnology, Bielefeld University, 33594 Bielefeld, Germany*¨

*Corresponding author: Miroslav Pátek e-mail: patek@biomed.cas.cz

Submitted to AMB Express

Table S1 Oligonucleotide primers used

Primer	Sequence[a]	Use
PRSHAPEPRF	*G*TCCCGACTCCATCGTGGAAGAAAACAGCTCCG AGGAATGTTAAAGGAAGTAGCGAAG*G*	P*rshA* cloning in pEPR1
PRSHAPEPRR	***G****A****T****CC*CTTCGCTACTTCCTTTAACATTCCTCGGAG CTGTTTTCTTCCACGATGGAGTCGGGA*C****TG****CA*	P*rshA* cloning in pEPR1
TRXB1F[b]	GT*C****TGCAG***ATCAATATCCACACCCT	P*trxB1*cloning in pEPR1
TRXB1R[b]	GCC*GG****ATCC***TGTGCTCTCAAATGT	P*trxB1*cloning in pEPR1
PSIGBHP[b]	CA***CTG****CA****G***AAGGTCACATCGGTT	P*sigB* cloning in pEPR1
PSIGBDB[b]	GT*GGA****TCC***TGCTGTCATAACTGG	P*sigB* cloning in pEPR1
PCG2556F[b]	ACA*C****TG****C****AG***AAATCTGGTTCAGGTATTC	P*cg2556* cloning in pEPR1
PCG2556R[b]	AAG*GG****A****TCC*TTCTTGTTTGCTG	P*cg2556* cloning in pEPR1
PCMT1PEPRF[c]	***G***TTCGAAAAGGTAAAGCGCCTGTTAACGTAATA GCTTGAAATATAGATGTAAATTAAA*G*	P*cmt1*cloning in pEPR1
PCMT1PEPRR[c]	*G****A****T****CC*TTTAATTTACATCTATATTTCAAGCTATTA CGTTAACAGGCGCTTTACCTTTTCGAA***C****T****GCA***	P*cmt1*cloning in pEPR1
PSIGAF[b]	AT***C****T****G****CAG*AAAGCACGAAAAGTG	P2*sigA* cloning in pEPR1

PSIGAR[b]	GGTG***GGATCC***CCTGGAAAGTCTCAT	P2*sigA* cloning in pEPR1
PFBAPEPRF[c]	***G***AGGAAATATCACACGACAAAAGTTGAGTGATG CAGGCATAATTGGCTATAGGCAACT*G*	P*fba* cloning in pEPR1
PFBAPEPRR[c]	***GATCC***AGTTGCCTATAGCCAATTATGCCTGCATC ACTCAACTTTTGTCGTGTGATATTTCCT***CTGCA***	P*fba* cloning in pEPR1
PRSHAP770F[c]	***AATTC***TCCCGACTCCATCGTGGAAGAAAACAGCTCC GAGGAATGTTAAAGGAAGTAGCGAAG***A***	P*rshA* cloning in pRLG770
PRSHAP770R[c]	***AGCTT***CTTCGCTACTTCCTTTAACATTCCTCGGAGCT GTTTTCTTCCACGATGGAGTCGGGA***G***	P*rshA* cloning in pRLG770
TRXB1P770F[c]	***AATTC***AGAAAATCCTTGGCCGGGAATAACTACAGT CCGCTGAAAGTTGGTCTATATATAGACC***A***	P*trxB1*cloning in pRLG770
TRXB1P770R[c]	***AGCTT***GGTCTATATATAGACCAACTTTCAGCGGACT GTAGTTATTCCCGGCCAAGGATTTTCT***G***	P*trxB1*cloning in pRLG770
PSIGBCGF[b]	GG***GAATTC***AGAACTCCATAAAAG	P*sigB* cloning in pRLG770
PSIGBCGR[b]	AT***AAGCTT***TACAAGAGGTTCAACGGAC	P*sigB* cloning in pRLG770
CG2556P770F[c]	*AATTC*ACCAATTCTTTAAAGCTCTCACCCTCTTTAGG GAACTGAATGCGGTCTGTACTCGACTACTGGATCAT GA***A***	P*cg2556* cloning in pRLG770
CG2556P770R[c]	***AGCTT***TCATGATCCAGTAGTCGAGTACAGACCGCAT TCAGTTCCCTAAAGAGGGTGAGAGCTTTAAAGAAT TGGT*G*	P*cg2556* cloning in pRLG770
PCMT1P770F[c]	*AATTC*GAAAAGGTAAAGCGCCTGTTAACGTAATAG CTTGAAATATAGATGTAAATTA*A*	P*cmt1*cloning in pRLG770
PCMT1P770R[c]	*AGCTT*TAATTTACATCTATATTTCAAGCTATTACGTT AACAGGCGCTTTACCTTTTC*G*	P*cmt1*cloning in pRLG770
PSIGAP770F[b]	AT***GAATTC***TCGAAAGGTGATTTTTGCC	P2*sigA* cloning in pRLG770
PSIGAP770R[b]	TCC***AAGCTT***AGCGTTCCATTATAGTTGA	P2*sigA* cloning in pRLG770
PFBAP770F[c]	***AATTC***AGGAAATATCACACGACAAAAGTTGAGTGA TGCAGGCATAATTGGCTATAGGCAACT***A***	P*fba* cloning in pRLG770
PFBAP770R[c]	***AGCTT***AGTTGCCTATAGCCAATTATGCCTGCATCAC TCAACTTTTGTCGTGTGATATTTCCT***G***	P*fba* cloning in pRLG770
SIGAPECF[b]	GC***GAATTCA***TCCTCAGCATCACTC	*sigA* cloning in pEC-XT99A
SIGAPECR[b]	TG***TCTAGA***CGAACCAAAGCAACAG	*sigA* cloning in pEC-XT99A
SIGBPECF[b]	AG***GAATTC***GTTGAACCTCTTGAAC	*sigB* cloning in pEC-XT99A
SIGBPECR[b]	GC***TCTAGA***ACTCGCCGGTAAAATA	*sigB* cloning in pEC-XT99A
SIGCPECF[b]	GGT***GAATTC***GGTCTGAACTGGTAT	*sigC* cloning in pEC-XT99A
SIGCPECR[b]	TTGA***TCTAGA***TCATCTGCTAACCTTGG	*sigC* cloning in pEC-XT99A
SIGDPECXTF[b]	CT***GAATTC***TAAACTTTTAGGGTTTTGATG	*sigD* cloning in pEC-XT99A

SIGDPECXTR[b]	GTC_**TCTAG**A_TTACTTGTTCTCCTGCTGCTC	*sigD* cloning in pEC-XT99A
EXSIGETCF1[c]	A**_AGAATTCGTCGAC_AG**G**AT**G**AGG**A**TC**G**TT**T**CGC**AT GAAAAAGAAGTCCCGAGATGACGCACCCG	*sigE* cloning in pEC-XT99A
EXSIGETCR1[b]	**AA**_**TCTAG**A_AAAGTTCCCTGTCCGCACCAC	*sigE* cloning in pEC-XT99A
EXSIGHTCF1[b]	**A**A_**GAATTCTGCAG**_ATAGTCAACACGCATTTTCGAAA GGGGC	*sigH* cloning in pEC-XT99A
EXSIGHTCR1[b]	**AA**_**TCTAGA**_CGAGTCGCTGCGGTTGAGA	*sigH* cloning in pEC-XT99A
EXSIGMTCF1[b]	**A**A_**GA**G**CTCTGCAG**_GCCCATACTAAGCCGCATAA	*sigM* cloning in pEC-XT99A
EXSIGMTCR1[b]	**AA**_**TCTAGA**_TCAAAGTAGTAAACGTAAAAGTGC	*sigM* cloning in pEC-XT99A

[a] sites for restriction enzymes are in italics, mismatched nucleotides are in bold
[b] oligonucleotide used for PCR cloning
[c] oligonucleotide used for direct association of complementary strands (labeled F and R) followed by cloning of the formed double stranded DNA fragments

Table S2 Identification of sigma factor σ^M by LC-MS/MS analysis. The table shows the sequence of the identified peptide, the corresponding Mascot score, m/z value and charge of the precursor ion used for MS/MS event, experimental and calculated molecular weight of the peptide, and mass error in ppm.

Peptide sequence	Mascot score	MS/MS precursor		Molecular weight		Error [ppm]
		m/z	Charge	Experimental	Calculated	
SAIDQLHPDQR	29	427.2175	3	1278.6307	1278.6317	0.77
LATDPLGYLDVAMTIR	49	874.9650	2	1747.9154	1747.9178	1.37
LATDPLGYLDVAMoxTIR	61	882.9614	2	1763.9082	1763.9128	2.56
KPEDAQDILQEALFR	35	591.6435	2	1771.9087	1771.9104	0.99
